# Scientometrics of peer review

**DOI:** 10.1007/s11192-017-2518-4

**Published:** 2017-09-09

**Authors:** Flaminio Squazzoni, Elise Brezis, Ana Marušić

**Affiliations:** 1 Department of Economics and Management, University of Brescia, Brescia, Italy; 2 Department of Economics, Bar-Ilan University, Ramat Gan, Israel; 3 Department of Research in Biomedicine and Health, University of Split School of Medicine, Split, Croatia

**Keywords:** Peer review, Scientometrics, Data, Interdisciplinary field

## Abstract

This article aims to introduce a special issue on “Scientometrics of peer review”, which collects papers originally presented at workshops and conferences organised by the COST ACTION TD1306 “New frontiers of peer review”. Peer review is the cornerstone of science and is one of the underlying processes that bring about publication traces that are at the heart of bibliometric studies. Unfortunately, despite its importance, quantitative studies on peer review are still poorly developed, often due to lack of data. The issue aims to promote the establishment of peer review as an interdisciplinary field of research and stimulate further quantitative research.

Peer review is the cornerstone of science, whose quality and efficiency depends on a complex, large-scale collaboration process. In case of Scientometrics and quantitative studies of science in general, peer review is one of the underlying processes that bring about publication traces that are at the heart of bibliometric studies. Indeed, peer review as a social process has attracted research interests also in the scientometrics community. For instance, research looked at the relationship of peer review and bibliometric indicators (Braun and Dióspatonyi 2005), the role of editors as gatekeepers in science (Nederhof and Raan 1987) and models of the peer review process (Ragone et al. 2013). This shows that although peer review as a field of cross-disciplinary research is still to be established, *Scientometrics* is an ideal publication venue for this research.

It is worth noting that this important institution of the science system has been recently under the spotlight not only in the academic debate but also in the public opinion. Recent testimonials of the failures of peer review, due to judgment bias and parochialism and cases of misconduct, as well as the explosion of online publications have contributed to calls for the reconsideration of the rigour and quality of the process (Casnici et al. 2017). Some analysts have questioned the lack of transparency and accountability of the process and stressed that there is poor systematic study on peer review, despite its importance in regulating resource allocation in science, e.g. funds, reputation and prestige. Recently, also thanks to online technologies, some journals have explored different models of peer review, i.e., releasing information on reviewers, supporting post-peer review experiments or providing reputational or material incentives to increase scientist commitment in the process. Unfortunately, there is lack of evidence against which to judge the implications of these changes.

This special issue aims to promote the establishment of peer review as an interdisciplinary field of research. Secondly, the availability of data from peer review, which is advocated by many observers, will probably enable us to have a better view of peer review and assessment processes in science in general (Lee and Moher 2017). In this respect, a noteworthy effort has been made within the framework of the COST Action “PEERE”—a group of researchers and publishers (i.e., Elsevier, Springer Nature and Wiley) who developed a protocol for sharing peer review data from many journals (Squazzoni et al. 2017).

The issue is supported by the COST ACTION TD1306 “New frontiers of peer review” (www.peere.org). This Action included various project meetings, including three workshops, respectively held at the Corvinus University of Budapest, October 2014, the University of Lisbon, January 2015, the ETH Zurich, March 2015, the University of Split, June 2015, at Athens, November 2015, at the University of Valencia, March 2016 and the Mykolas Romeris University in Vilnius, on March 2017. A selection of the best contributions was made among about 50 papers which have been presented. The COST Action has supported OA fees for all accepted articles.

## References

[CR1] Braun T, Dióspatonyi I (2005). The journal gatekeepers of major publishing houses of core science journals. Scientometrics.

[CR2] Casnici N, Grimaldo F, Gilbert N, Squazzoni F (2017). Attitudes of referees in a multidisciplinary journal: An empirical analysis. Journal of the Association for Information Science and Technology.

[CR3] Lee CJ, Moher D (2017). Promote scientific integrity via journal peer-review data. Science.

[CR4] Nederhof AJ, Raan AFJ (1987). Peer review and bibliometric indicators of scientific performance: A comparison of cum laude doctorates with ordinary doctorates in physics. Scientometrics.

[CR5] Ragone A, Mirylenka K, Casati F, Marchese M (2013). On peer review in computer science: Analysis of its effectiveness and suggestions for improvement. Scientometrics.

[CR6] Squazzoni F, Grimaldo F, Marušić A (2017). Journals could share peer-review data. Nature.

